# Association between DNA methylation predicted growth differentiation factor 15 and mortality: results from NHANES 1999–2002

**DOI:** 10.1007/s40520-024-02896-3

**Published:** 2024-12-03

**Authors:** Honglian Luo, Yun Shen

**Affiliations:** 1 Tongji Medical College, Huazhong University of Science and Technology, Wuhan, 430030 China; 2 Pennington Biomedical Research Center, Baton Rouge, LA 70808 USA

**Keywords:** NHANES database, DNA methylation, GDF-15, All-cause mortality, Cardiovascular mortality

## Abstract

**Background:**

Growth differentiation factor 15 (GDF15) is a crucial biomarker in various physiological and pathological processes. While elevated GDF15 levels are linked to increased mortality risk, the role of DNA methylation (DNAm)-predicted GDF15 in predicting mortality has not been extensively studied. The purpose of the study is to investigate the association between DNAm-predicted GDF15 levels and all-cause and cardiovascular disease (CVD) mortality in a nationally representative cohort.

**Methods:**

Data from NHANES 1999–2002 were analyzed. DNAm-predicted GDF15 levels were estimated using a regression model. Weighted multivariate Cox regressions were employed to assess the relationship between DNAm-predicted GDF15 and mortality outcomes. Restricted cubic splines were used to explore dose-response relationships, and subgroup analyses were conducted to enhance result reliability.

**Results:**

Higher DNAm-predicted GDF15 levels were significantly associated with increased all-cause mortality risk (HR = 1.08, 95% CI: 1.02–1.15). Participants in the highest DNAm-predicted GDF15 tertile showed significantly higher all-cause mortality risk (HR = 1.56, 95% CI: 1.16–2.10) and a 2.52-fold increased risk of cardiovascular mortality (HR = 2.52, 95% CI: 1.22–5.19). Kaplan-Meier curves revealed decreasing survival probability with higher DNAm-predicted GDF15 tertiles. Restricted cubic spline analysis demonstrated a non-linear dose-response relationship between DNAm-predicted GDF15 levels and cardiovascular mortality. The positive correlation between DNAm-predicted GDF15 and mortality remained robust in most of subgroups.

**Conclusions:**

DNAm-predicted GDF15 independently predicts all-cause and cardiovascular mortality. This association persists across multiple models and stratified subgroups, supporting GDF15’s value as a biomarker for mortality risk stratification. Future research should elucidate underlying biological mechanisms and evaluate GDF15’s clinical utility in guiding mortality risk reduction interventions.

## Introduction

Growth differentiation factor 15 (GDF15), a component of the Transforming growth factor β (TGF-β) superfamily, has gained significant attention in recent years. Researchers have identified it as a crucial molecular indicator in a range of physiological and pathological conditions, such as inflammatory responses, cellular stress reactions, and tissue injury [1–4]. Elevated levels of GDF15 have been linked to a range of adverse health outcomes [5–7], where it is often considered a marker of increased mortality risk. Several studies have explored its role in predicting mortality in patients with cardiovascular disease, cancer, and chronic illnesses, highlighting the importance of GDF15 in prognostic models. However, the molecular mechanisms underpinning GDF15 expression and its direct impact on mortality remain poorly understood.

Recent advances in epigenetics have revealed that DNA methylation (DNAm), an essential regulator of gene expression, may influence the levels of circulating biomarkers [8, 9]. DNA methylation can modulate gene expression and has been increasingly studied as a predictor of disease risk and mortality. By predicting GDF15 levels through DNA methylation signatures, it may be possible to provide novel insights into the role of GDF15 in mortality and enhance risk prediction models. The NHANES dataset, with its extensive information and linked dataset with national death registry, offers a unique opportunity to study the link between DNAm-predicted GDF15 and mortality. This research aimed to explore the potential link between DNAm-predicted GDF15 levels and both all-cause and cardiovascular mortality.

## Methods

### Study population

**Fig. 1:**
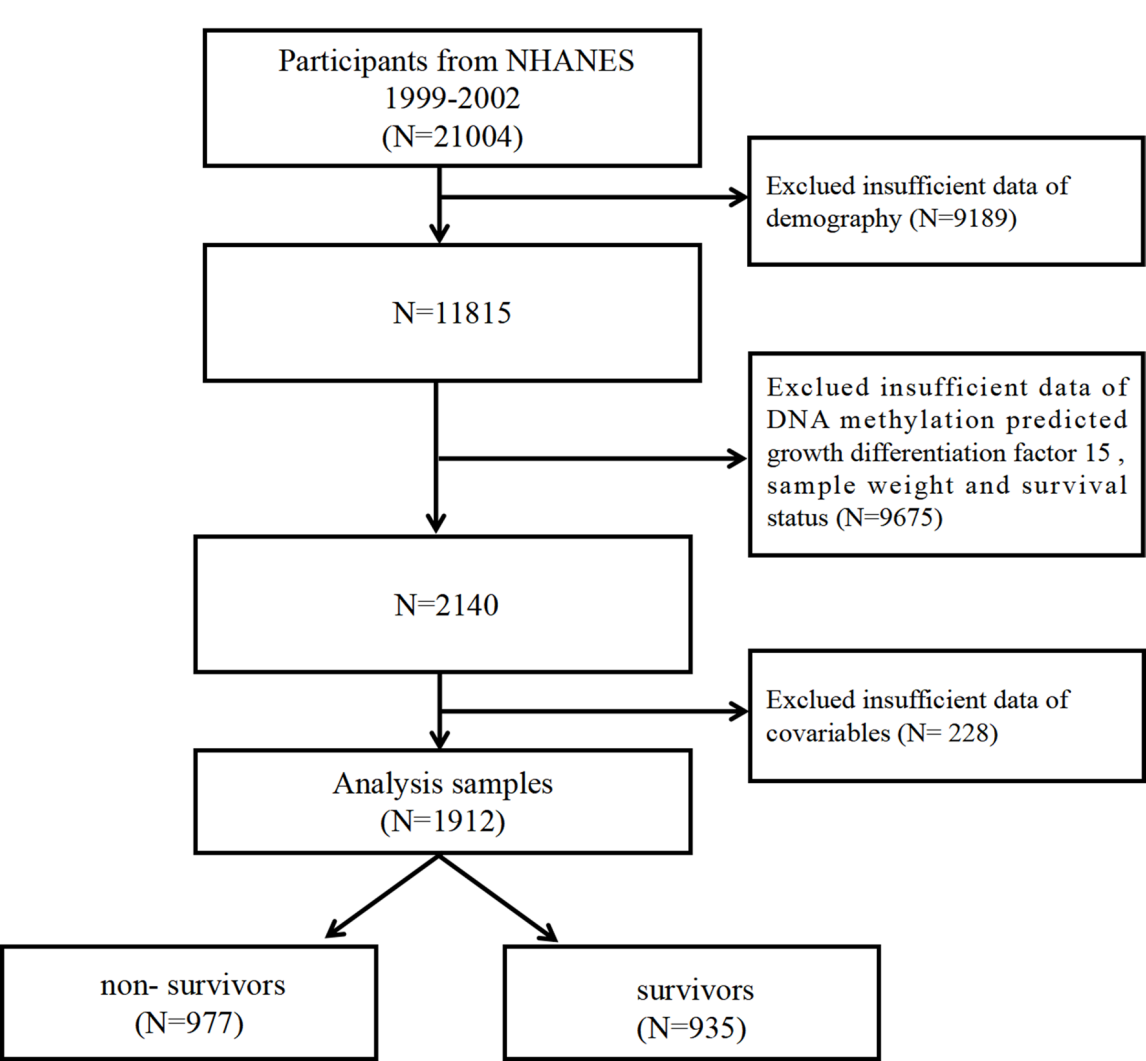
Flow chart of the study participants’ selection process

The NHANES database provides all information of the research, which is collected through a nationwide cross-sectional study carefully designed and executed by the Center for Disease Control and Prevention (CDC) to closely observe the health and nutritional condition of the population in the United States [10, 11]. Our study encompassed data collected across two NHANES cycles, spanning from 1999 to 2002, as only these two survey cycles provided complete data on DNA methylation. A total of 21,004 individuals participated in the NHANES 1999 to 2002 survey. We excluded participants with insufficient data on demographics (*N* = 9,189), insufficient data on DNA methylation and sample weights (*N* = 9,675), and insufficient data on covariables (*N* = 228). The final analytic dataset comprised 1912 participants with 977 of them deceased and 935 survived (Fig. 1). The NCHS Ethics Review Board approved the NHANES data collection (Protocols #98 − 12), and all participants provided written informed consent. Furthermore, the design and report of the research adhered to the guidelines in the Declaration of Helsinki and the STROBE Statement [12].

### Data collection

The NHANES 1999–2002 cycles employed a complex, multistage probability sampling design to represent the U.S. civilian non-institutionalized population. Data collection included in-home interviews and physical examinations at mobile examination centers (MECs). Demographic, socioeconomic, dietary, and health-related information was collected through structured interviews administered by trained interviewers. Physical examinations included measurements of height, weight, blood pressure, and other clinical assessments. Biological samples were collected for laboratory analysis. All data extracted from the database in this analysis included age, sex, ethnicity, marital status, poverty-to-income ratio (PIR), body mass index (BMI), alcohol consumption, daily energy intake, education level, smoking status, C-reactive protein, total cholesterol (TC) and history of comorbidities (including cardiovascular disease (CVD), chronic kidney disease (CKD), hypertension, diabetes mellitus, and cancer).

### Eligible samples and DNA methylation

In the NHANES 1999–2002 survey cycles, adults aged 50 years and over who had blood collected for DNA purification, were eligible. The sample comprises roughly half of the eligible non-Hispanic White participants selected randomly, along with all eligible participants from non-Hispanic Black, Mexican American, other Hispanic, and other racial groups. DNA was extracted from the whole blood sample and stored at -80°C. The DNAm assay was conducted in Dr. Yongmei Liu’s laboratory at Duke University. Bisulfite conversion of DNA followed the manufacturer’s guidelines. DNA (500ng) underwent bisulfite conversion using Zymo’s EZ Methylation kit. The converted DNA was then amplified following Illumina’s Infinium assay PCR protocol (16 cycles: 95°C/30s, 50°C/60min). Subsequently, 4 µL of bisulfite-converted DNA was processed using Illumina’s Infinium HD Methylation protocol. Steps included overnight denaturation and amplification (20-24 h), fragmentation, precipitation, resuspension, and EPIC BeadChip v1.0 hybridization (16-24 h). After washing, nucleotide labeling extended primers. The BeadChip was then imaged via Illumina’s iScan system to obtain methylation data. To preprocess and analyze NHANES blood samples, a regression calibration algorithm was employed to predict cell type proportions from DNAm data. The IDOL probe subset and the FlowSorted.Blood.EPIC_ref reference dataset was utilized on NHANES data to predict cell type proportions using the ‘estimateCellCounts2’ function from the immunomethylomics/FlowSorted.Blood.EPIC package [13–15]. DNAm-predicted GDF15 values were estimated by regression on chronological age, sex, and the relevant CpGs levels.

### All-cause and cardiovascular mortality

Mortality status was ascertained by linking NHANES participant records to the National Death Index (NDI). The duration of participants’ follow-up could be determined by calculating the time interval between the baseline survey date and either the date of the most recent mortality status update or the last update date of the NDI database (on December 31, 2019), whichever occurred first [16]. The primary outcomes of interest were all-cause mortality and cardiovascular mortality. The International Classification of Diseases, 10th Revision (ICD-10) was employed to identify mortality causes. The cardiovascular mortality was defined as I20–I51, I13, I11 and I00–I09 [17].

### Statistical analysis

Statistical analyses were conducted using the survey procedures to account for the complex sampling design of NHANES. Since it was a subset of data from the overall sample, specific sample weights were used in the analysis. Weighted Cox proportional hazards models were used to estimate hazard ratios (HRs) and 95% confidence intervals (CIs) for the association between DNAm-predicted GDF15 and mortality outcomes. Model 1 did not include any covariate adjustments. Model 2 accounted for ethnicity, sex, and age. Model 3 enhanced Model 2 by incorporating extra covariates: education level, PIR, marital condition, smoking and alcohol status, total energy intake, CRP, BMI, total cholesterol, and history of comorbidities (hypertension, diabetes, cardiovascular disease, chronic kidney disease, and cancer). To enhance the robustness of the research, the DNAm-predicted GDF15 was transformed into a categorical variable using tertiles, which were marked as T1-T3 serially. To further visualize the association between DNAm-predicted GDF15 levels and survival, we performed Kaplan-Meier survival analysis to estimate survival probabilities across different tertiles of DNAm-predicted GDF15. Kaplan-Meier survival curves were plotted to compare the cumulative probability of survival over time among the different tertiles. A restricted cubic spline analysis was conducted to examine the dose-response relationship between DNAm-predicted GDF15 and mortality. Subgroup analyses were conducted, stratified by age (< 65 or ≥ 65), BMI (< 25 or ≥ 25 kg/m^2^), sex (male or female), ethnic categories, poverty-to-income ratio (PIR < 1.3 or 1.3–3.5 or > 3.5), educational background (less than high school or high school or more than high school), smoking status (former smoker or current smoker or never smoking), alcohol consumption (never; former; mild; moderate; heavy), diabetes (No or Yes), hypertension (No or Yes), cardiovascular disease (No or Yes), chronic kidney disease (No or Yes), and cancer (No or Yes). Furthermore, these stratified factors were considered as predetermined potential modifiers of the effects. To assess variability in associations across subgroups, interaction terms were included, and likelihood ratio tests were employed. R version 4.3.3 was used for all analyses and generation of figures (R Foundation for Statistical Computing, Vienna, Austria). A *P*-value less than 0.05 was considered to be statistically significant.

## Results

### Baseline characteristics of participants

The study included a total of 1,912 individuals meeting inclusion and exclusion criteria. Table 1 summarizes the demographic baseline characteristics of the study population based on the survival status. Those who deceased had a higher mean age (70.23 ± 0.54 years) compared to those who survived (58.33 ± 0.33 years). In contrast, the mean BMI did not differ significantly between survivors (28.80 ± 0.31 kg/m^2^) and non-survivors (28.33 ± 0.26 kg/m^2^). The C-reactive protein was significantly higher in non-survivors (0.66 ± 0.08 mmHg) compared to survivors (0.43 ± 0.02 mmHg). Total cholesterol levels showed no statistically significant difference between survivors (5.50 ± 0.05 mmol/L) and non-survivors (5.46 ± 0.05 mmol/L, *P* = 0.42). Energy intake was significantly lower in non-survivors (1762.18 ± 31.15 kcal) compared to survivors (2016.49 ± 26.87 kcal). The comorbidities, including diabetes, hypertension, cardiovascular diseases, chronic kidney disease and cancer, differed significantly between those who deceased and those who survived. Refer to Table 1 for additional information.

**Table 1:** Baseline characteristics of the study population from NHANES 1999–2002

Variable	Survived *N* = (935)	Deceased *N* = (977)	*P* value
Age, years	58.33(0.33)	70.23(0.54)	< 0.001
DNAm-predicted GDF15, ng/L	853.59(6.22)	1031.05(7.14)	< 0.001
Body Mass Index, kg/m^2^	28.80(0.31)	28.33(0.26)	0.12
Total cholesterol, mmol/L	5.50(0.05)	5.46(0.05)	0.42
Daily energy intake, kcal	2016.49(26.87)	1762.18(31.15)	< 0.001
C-reactive protein, mg/dL	0.43(0.02)	0.66(0.08)	0.01
**Sex**,** n (%)**			0.43
Female	485(55.63)	450(52.89)	
Male	450(44.37)	527(47.11)	
**Ethnicity**,** n (%)**			0.08
Mexican American	300(3.85)	231(2.72)	
Non-Hispanic Black	165(6.80)	217(8.67)	
Non-Hispanic White	362(79.63)	457(81.34)	
Other Hispanic	69(6.05)	47(4.73)	
Other ethnicity	39(3.68)	25(2.54)	
**Marital status**,** n (%)**			0.004
Not married	272(26.28)	390(36.85)	
Married or living with partner	663(73.72)	587(63.15)	
**Education level**,** n (%)**			< 0.001
< High school	186(4.86)	281(13.74)	
High school	339(37.18)	425(51.10)	
> High school	410(57.95)	271(35.16)	
**PIR**,** n (%)**			< 0.001
< 1.3	217(14.24)	328(25.65)	
1.3–3.5	315(26.51)	429(45.15)	
> 3.5	403(59.25)	220(29.20)	
**Smoking status**,** n (%)**			0.38
Never	462(45.68)	418(41.41)	
Former	354(39.52)	404(41.90)	
Now	119(14.80)	155(16.69)	
**Alcohol consumption**,** n (%)**			< 0.001
Never	144(12.94)	181(17.86)	
Former	212(18.76)	330(35.04)	
Mild	365(42.85)	311(33.58)	
Moderate	101(14.51)	79(8.09)	
Heavy	113(10.94)	76(5.43)	
**Hypertension**,** n (%)**			< 0.001
No	450(52.63)	254(27.93)	
Yes	485(47.37)	723(72.07)	
**Diabetes**,** n (%)**			< 0.001
No	774(89.94)	695(75.92)	
Yes	161(10.06)	282(24.08)	
**CVD**,** n (%)**			< 0.001
No	841(90.22)	707(70.55)	
Yes	94(9.78)	270(29.45)	
**CKD**,** n (%)**			< 0.001
No	799(87.35)	550(57.58)	
Yes	136(12.65)	427(42.42)	
**Cancer**,** n (%)**			< 0.001
No	846(88.05)	799(77.09)	
Yes	89(11.95)	178(22.91)	

### The association between DNA methylation-predicted GDF15 and all-cause mortality

The association between DNAm-predicted GDF15 and all-cause mortality was significant in all models (Table 2). In the unadjusted model (Model 1), DNAm-predicted GDF15 as a continuous variable was associated with a hazard ratio (HR) of 1.48 (95% CI: 1.36–1.61, *P* < 0.001), and as a categorical variable, the second tertile (T2) had an HR of 3.17 (95% CI: 2.39–4.21, *P* < 0.001), while the third tertile (T3) showed an HR of 9.17 (95% CI: 6.97–12.08, *P* < 0.001), with a significant trend (*P* for trend < 0.001). After adjusting for age, sex, and ethnicity in Model 2, the HR for GDF15 as a continuous variable decreased to 1.17 (95% CI: 1.09–1.26, *P* < 0.001). Similarly, the HRs for T2 and T3 were reduced to 1.55 (95% CI: 1.17–2.06, *P* = 0.003) and 2.36 (95% CI: 1.70–3.28, *P* < 0.001), respectively, with a significant trend (*P* for trend < 0.001). In the fully adjusted model (Model 3), which controlled for additional covariates including marital status, socioeconomic status, and various health measures, the HR for GDF15 as a continuous variable further decreased to 1.08 (95% CI: 1.02–1.15, *P* = 0.01), while T2 and T3 had HRs of 1.21 (95% CI: 0.94–1.56, *P* = 0.14) and 1.56(95% CI: 1.16–2.10, *P* = 0.003), with the trend remaining significant (*P* for trend = 0.003).

**Table 2:** Hazard ratios of DNAm-predicted GDF15 in association with all-cause and cardiovascular mortality

All-cause mortality	Model 1			Model 2		Model 3	
	HR (95% CI)		*P*	HR (95% CI)	*P*	HR (95% CI)	*P*
DNAm-predicted GDF15 as continuous variable							
Per 100 units	1.48(1.36,1.61)	< 0.001		1.17(1.09,1.26)	< 0.001	1.08(1.02,1.15)	0.01
DNAm-predicted GDF15 as categories variable (T1 as reference)							
T2	3.17(2.39, 4.21)	< 0.001		1.55(1.17,2.06)	0.003	1.21(0.94,1.56)	0.14
T3	9.17(6.97,12.08)	< 0.001		2.36(1.70,3.28)	< 0.001	1.56(1.16,2.10)	0.003
P for trend		< 0.001			< 0.001		0.003
**Cardiovascular mortality**							
DNAm-predicted GDF15 as continuous variable							
Per 100 units	1.52(1.37,1.68)	< 0.001		1.19(1.05,1.36)	0.01	1.14(1.00,1.31)	0.05
DNAm-predicted GDF15 as categories variable (T1 as reference)							
T2	6.84(4.27,10.96)	< 0.001		3.13(1.88,5.22)	< 0.001	2.36(1.44,3.84)	< 0.001
T3	15.47(9.49,25.20)	< 0.001		3.58(1.78,7.22)	< 0.001	2.52(1.22,5.19)	0.01
P for trend		< 0.001			0.01		0.06

Model 1: Not adjusted any covariates; Model 2: Adjusted age, sex and ethnicity; Model 3: Adjusted age, sex, ethnicity, marital status, poverty-to-income ratio, education level, smoking status, alcohol status, daily energy intake, CRP, BMI, total cholesterol, history of comorbidities (hypertension, diabetes, CVD, CKD and cancer)

### The association between DNAm-predicted GDF15 and cardiovascular mortality

For cardiovascular mortality, DNAm-predicted GDF15 as a continuous variable was associated with a significant increase in risk in Model 1, with an HR of 1.52 (95% CI: 1.37–1.68, *P* < 0.001) (Table 2). In Model 2, after adjusting for age, sex, and ethnicity, the HR decreased to 1.19 (95% CI: 1.05–1.36, *P* = 0.01), but in the fully adjusted model (Model 3), the association was no longer significant (HR = 1.14, 95% CI: 1.00–1.31, *P* = 0.05). As a categorical variable, T2 had an HR of 6.84 (95% CI: 4.27–10.96, *P* < 0.001) in Model 1, which decreased to 3.13 (95% CI: 1.88–5.22, *P* < 0.001) in Model 2, and to 2.36 (95% CI: 1.44–3.84, *P* < 0.001) in Model 3. For T3, the HR was 15.47 (95% CI: 9.49–25.20, *P* < 0.001) in Model 1, 3.58 (95% CI: 1.78–7.22, *P* < 0.001) in Model 2, and 2.52 (95% CI: 1.22–5.19, *P* = 0.01) in Model 3 (*P* for trend = 0.06).

### Kaplan-Meier survival analysis

**Fig. 2:**
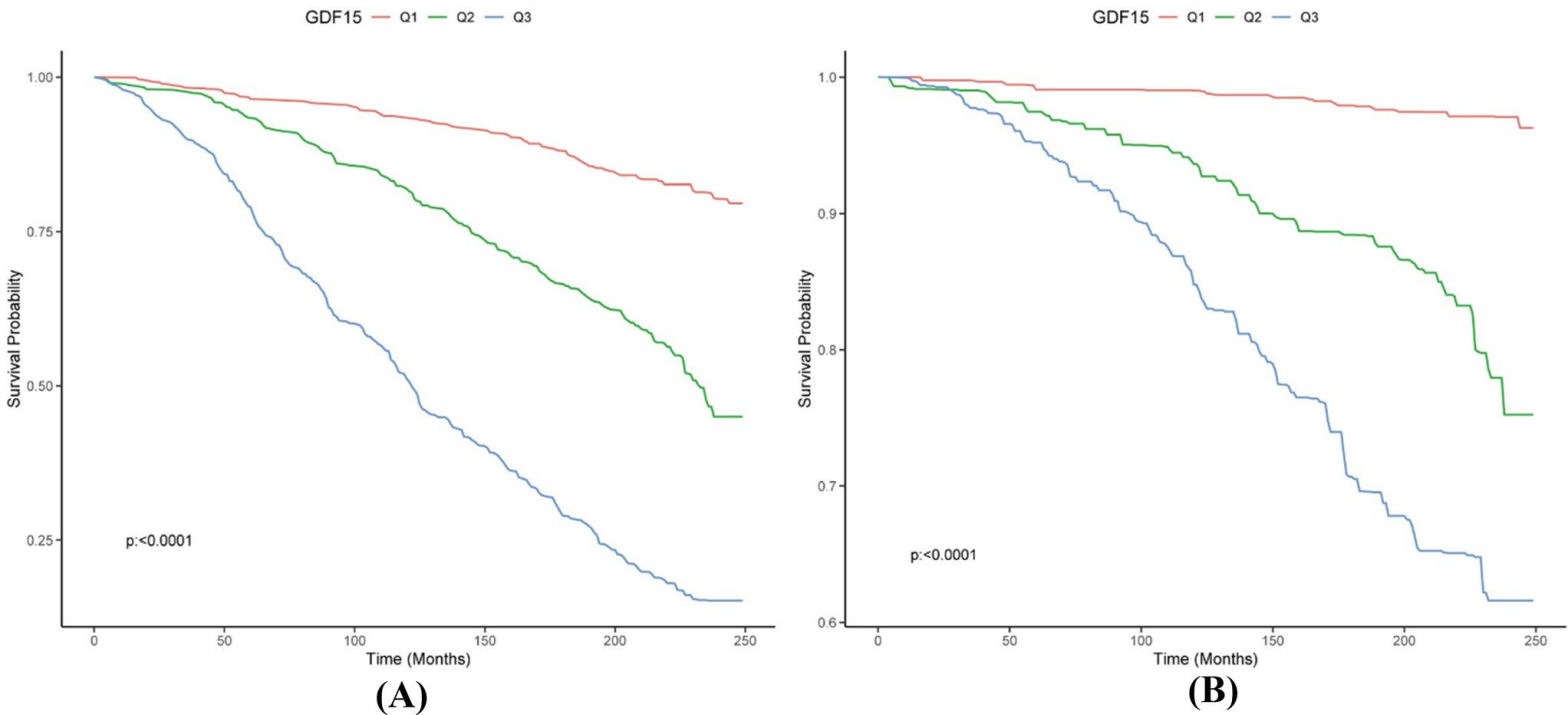
Kaplan-Meier survival curves showed the cumulative probability of survival over time among the different tertiles. (**A**) All-cause mortality was designed as the clinical endpoint; (**B**) Cardiovascular mortality was designed as the clinical endpoint

The Kaplan-Meier survival curves (Fig. 2) revealed a significant difference in all-cause survival probability among the DNAm-predicted GDF15 tertiles (T1, T2, and T3) over time (*P* < 0.0001). Survival probability progressively decreases with higher tertiles of GDF15 compared to the lowest tertile (T1). The findings were consistent when we used cardiovascular mortality as the outcome.

### Exploring the non-linear association of DNAm-predicted GDF15 and mortality

**Fig. 3:**
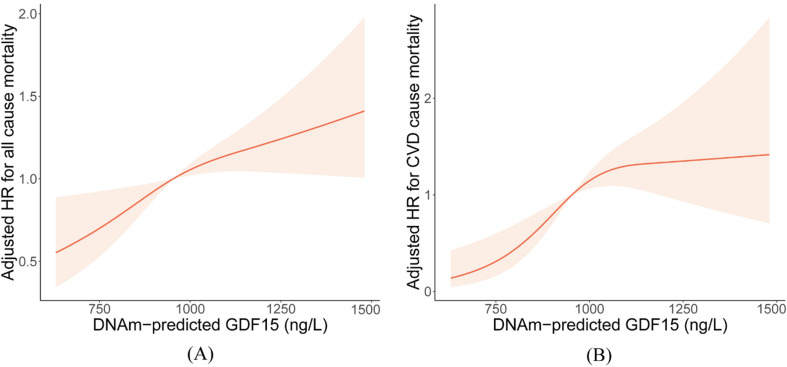
Exploring the potential non-linear association of DNAm-predicted GDF15 and mortality. (**A**) RCS when outcome was designed as all-cause mortality; (**B**) RCS when outcome was designed as cardiovascular mortality

We used a restricted cubic spline plot (Fig. 3) to exploring the dose-response association between DNAm-predicted GDF15 and mortality. The association between GDF15 levels and the adjusted HR for all-cause mortality was linear (*P* for overall < 0.05; *P* for nonlinearity > 0.05), whereas the association with cardiovascular mortality was non-linear (*P* for overall < 0.05; *P* for nonlinearity < 0.05). Increasing DNAm-predicted GDF15 levels were associated with a gradual rise in the hazard ratio for cardiovascular mortality. However, once DNAm-predicted GDF15 levels exceeded approximately 1000ng/L, the rate of increase became more gradual.

### Subgroup analysis

**Fig. 4:**
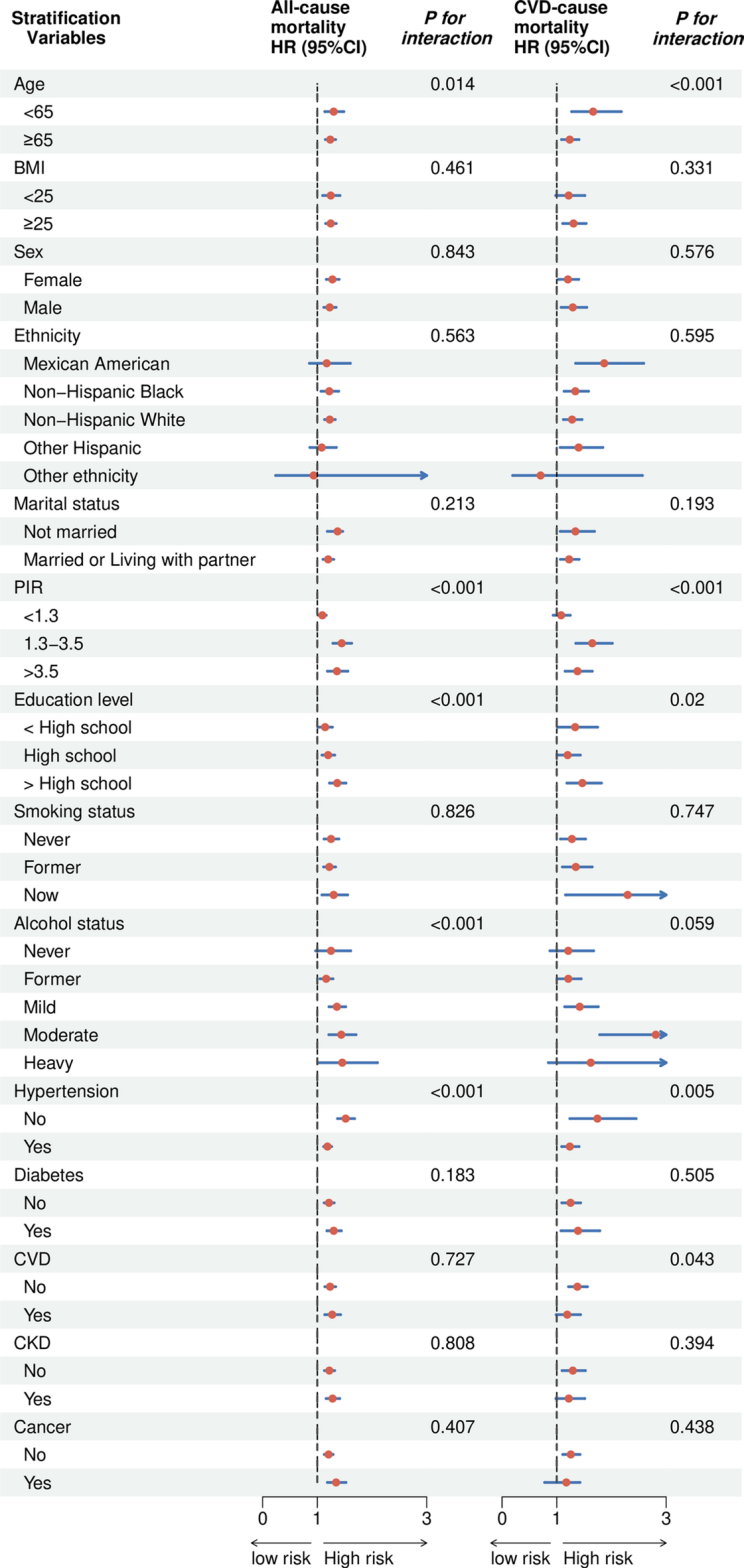
The Forest plot showed the correlation between DNAm-predicted GDF15 and mortality (HR 95%)

We further investigated the robustness of the findings in different subgroups (Fig. 4). The association between DNAm-predicted GDF15 and all-cause mortality was significant across multiple stratified subgroups including age, PIR, education levels and alcohol consumption with a significant interaction effect. In terms of ethnicity, DNAm-predicted GDF15 was significantly associated with mortality across all ethnicity subgroups, with no significant interaction (*P* for interaction = 0.563). Similarly, BMI, sex and smoking status did not show significant interactions (all *P* for interaction > 0.05) with DNAm-predicted GDF15 in predicting mortality, However, DNAm-predicted GDF15 was a significant predictor in all of the above subgroups. DNAm-predicted GDF15 remained a significant predictor of mortality across individuals with or without hypertension, diabetes, cardiovascular disease, chronic kidney disease, or cancer. Similar results could also be found when cardiovascular mortality was considered as the outcome.

## Discussion

In this study using the NHANES 1999–2002 data, we demonstrated that a significant association between DNAm-predicted GDF15 and all-cause and cardiovascular mortality. Our findings demonstrated that higher levels of DNAm-predicted GDF15 were strongly associated with an increased risk of mortality, independent of demographic, lifestyle, and clinical factors. These associations persisted across multiple models and stratified subgroups, providing further evidence of the potential role of DNAm-predicted GDF15 as a key biomarker for mortality risk.

Several studies have found a strong association between elevated GDF15 levels and all-cause mortality while based on direct measurements from serum or plasma samples. Bao et al. found that GDF-15 was a robust biomarker for all-cause mortality based on a cohort study up to 20 years of follow-up [18]. Another study from Japan also showed that GDF15 could predict cancer mortality among patients with cardiovascular risk factors [19]. However, findings might be inconsistent among different populations. Binder et al. reported that GDF15 levels were linked to heart failure hospitalization and mortality, but not to ventricular arrhythmic events [20]. The research conducted by Freeman et al. indicated that, poverty status modified the effect of elevated GDF15 on all-cause mortality, revealing a significant interaction between GDF15 and poverty status on all-cause mortality [21]. While these studies supported the strong association between GDF15 and mortality, few studies considered a more precise estimate of GDF15 based on DNA methylation. We provided robust evidence linking DNA methylation-predicted GDF15 with all-cause and cardiovascular mortality.

GDF15 is increasingly recognized as a stress-responsive cytokine with diverse roles in cellular metabolism [22], inflammation [23], mitochondrial function [24], and systemic stress responses [25]. Recent studies indicated that GDF15 expression was upregulated in response to mitochondrial toxins and mitochondrial DNA mutations, suggesting that it acted as a stress-responsive cytokine that signaled mitochondrial impairment to surrounding tissues [26, 27]. GDF15 was also regulated by key mitochondrial proteins including peroxisome proliferator-activated receptor gamma coactivator 1 alpha (PGC-1α) and activating transcription factor 4 (ATF4), which were involved in mitochondrial biogenesis and cellular response to oxidative stress [27, 28]. Under oxidative stress, GDF15 could be upregulated and could activate the nuclear factor erythroid 2-related factor 2 (NRF2) pathway to start antioxidant defenses [29]. Under prolonged mitochondrial dysfunction and oxidative stress, GDF15 could also activate apoptotic pathways, including the intrinsic mitochondrial apoptosis pathway, leading to the release of cytochrome c and activation of caspases. Chronic low-grade mitochondrial stress may trigger GDF-15-mediated apoptosis as a means of removing damaged cells to maintain tissue integrity. Moreover, GFRAL has been shown to be the receptors of GDF15, modulating inflammatory and apoptotic responses in stressed cells, which is significant for its potential as a therapeutic target.

GDF15 is also produced in response to cellular stress, particularly in situations of tissue damage, hypoxia, or oxidative stress. It is highly expressed in various tissues, including the heart, liver, and immune cells, in response to pro-inflammatory stimuli such as interleukin-6 (IL-6) and tumor necrosis factor-alpha (TNF-α) [30, 31]. In addition, GDF15 has been shown to modulate key inflammatory pathways by inhibiting the activation of macrophages and reducing the production of pro-inflammatory cytokines. This may represent a compensatory mechanism to limit excessive inflammation. However, chronic elevation of GDF15, as seen in aging and chronic diseases [32, 33], is thought to reflect ongoing systemic inflammation, which is a well-known contributor to all-cause and cardiovascular mortality. Chronic low-grade inflammation, also referred to as “inflammaging,” is a hallmark of aging and has been linked to an increased risk of mortality. Elevated GDF15 levels could signal the presence of such chronic inflammation, contributing to an elevated risk of mortality in older individuals.

Frailty is an important predictor of mortality, especially in older adults. GDF15 has been identified as a biomarker of frailty due to its role in signaling physiological stress, including muscle atrophy and reduced physical function [34]. Elevated levels of GDF15 in frail individuals may reflect an accumulation of stress-related damage and a diminished ability to cope with metabolic and inflammatory challenges, leading to an increased risk of mortality. Other critical mechanisms may include the responses of GDF15 to mitochondrial dysfunction, oxidative stress, cellular stress and apoptosis. DNA methylation at specific CpG sites can alter gene expression, and methylation patterns may change in response to environmental stressors, aging, and disease states. It is likely that epigenetic modifications influence GDF15’s expression, reflecting an individual’s cumulative exposure to stress and disease risk factors. DNA methylation changes associated with aging and chronic diseases may increase the expression of GDF15, contributing to the increased risk of mortality observed in older adults and individuals with comorbidities [35–37]. This epigenetic regulation may provide a more stable and long-term measure of physiological stress than circulating levels of GDF15 alone.

We also observed significant interactions between individuals with different ages, PIR, education, and alcohol consumption levels, as well as individuals with or without hypertension at baseline. The observed interactions between DNA methylation-predicted GDF15 levels and various demographic and health-related factors suggested that the impact of GDF15 on mortality risk may differ depending on individual characteristics. For example, individuals with excessive alcohol consumptions are more vulnerable to the adverse effects of high GDF15, as the condition already involve chronic inflammation and vascular stress. Similarly, our findings regarding the interaction with PIR are consistent with previous research in urban African American and white adults, which demonstrated that socioeconomic disparities might modify the association between GDF15 and mortality risk [21]. While similar interactions were observed with other demographic and clinical characteristics, further research is needed to elucidate the biological mechanisms underlying these differential associations.

The strengths of this study lie in the use of DNAm-predicted GDF15 levels, which provide a more objective measure of long-term biomarker exposure, and the use of a large, nationally representative cohort with long-term follow-up. The use of multiple models and sensitivity analyses across various subgroups enhances the validity of our findings. However, several limitations should be fully addressed. First, in this observational study, while we identified a significant association between DNAm-predicted GDF15 levels and mortality, it is important to recognize that this design inherently limits our ability to establish causality. Reverse causation is a potential concern, where underlying health conditions could both elevate GDF15 levels and contribute to mortality risk. To address these causality concerns, future research could incorporate longitudinal study designs, which allow for the observation of changes in GDF15 levels over time in relation to mortality outcomes. Longitudinal studies or mendelian randomization analyses would provide insight into the temporal relationship between GDF15 and mortality, helping to clarify whether changes in GDF15 levels precede increased mortality risk or arise as a consequence of declining health. Secondly, our study focused on a cohort from NHANES 1999–2002, and future studies should examine whether these findings are generalizable to more recent cohorts or other populations. Additionally, residual confounding may influence the observed association, as factors not fully accounted for in our analysis, such as lifestyle factors, genetic predispositions, or other biomarkers, could contribute to mortality outcomes.

## Conclusion

In conclusion, DNAm-predicted GDF15 was a strong, independent predictor of all-cause and cardiovascular mortality. Its association with mortality was consistent across most of subgroups, making it a valuable biomarker for risk stratification. Future research should focus on the potential mechanisms underlying the relationship between GDF15 and mortality and explore its clinical utility in guiding interventions aimed at reducing mortality risk.

## Data Availability

All data are publicly accessible at https://www.cdc.gov/nchs/nhanes/.

## Abbreviations

GDF15: Growth differentiation factor 15
DNAm: DNA methylation
CVD: Cardiovascular disease
RCS: Restricted cubic spline
NHANES: National Health and Nutrition Examination Survey
CRP: C-reactive protein
BMI: Body mass index
PIR: Poverty to income ratio
TC: Total cholesterol
CKD: Chronic kidney disease
CI: Confidence interval
